# A kirigami-enabled electrochromic wearable variable-emittance device for energy-efficient adaptive personal thermoregulation

**DOI:** 10.1093/pnasnexus/pgad165

**Published:** 2023-06-13

**Authors:** Ting-Hsuan Chen, Yaoye Hong, Ching-Tai Fu, Ankita Nandi, Wanrong Xie, Jie Yin, Po-Chun Hsu

**Affiliations:** Thomas Lord Department of Mechanical Engineering and Materials Science, Duke University, Durham, NC 27708, USA; Department of Mechanical and Aerospace Engineering, North Carolina State University, Raleigh, NC 27695, USA; Thomas Lord Department of Mechanical Engineering and Materials Science, Duke University, Durham, NC 27708, USA; Ming Hsieh Department of Electrical and Computer Engineering, University of Southern California, Los Angeles, CA 90089, USA; Thomas Lord Department of Mechanical Engineering and Materials Science, Duke University, Durham, NC 27708, USA; Department of Applied Physics and Materials Science, California Institute of Technology, Pasadena, CA 91125, USA; Thomas Lord Department of Mechanical Engineering and Materials Science, Duke University, Durham, NC 27708, USA; Department of Applied Physical Sciences, University of North Carolina at Chapel Hill, Chapel Hill, NC 27599, USA; Department of Mechanical and Aerospace Engineering, North Carolina State University, Raleigh, NC 27695, USA; Thomas Lord Department of Mechanical Engineering and Materials Science, Duke University, Durham, NC 27708, USA; Pritzker School of Molecular Engineering, The University of Chicago, Chicago, IL 60637, USA

## Abstract

For centuries, people have put effort to improve the thermal performance of clothing to adapt to varying temperatures. However, most clothing we wear today only offers a single-mode insulation. The adoption of active thermal management devices, such as resistive heaters, Peltier coolers, and water recirculation, is limited by their excessive energy consumption and form factor for long-term, continuous, and personalized thermal comfort. In this paper, we developed a wearable variable-emittance (WeaVE) device, enabling the tunable radiative heat transfer coefficient to fill the missing gap between thermoregulation energy efficiency and controllability. WeaVE is an electrically driven, kirigami-enabled electrochromic thin-film device that can effectively tune the midinfrared thermal radiation heat loss of the human body. The kirigami design provides stretchability and conformal deformation under various modes and exhibits excellent mechanical stability after 1,000 cycles. The electronic control enables programmable personalized thermoregulation. With less than 5.58 mJ/cm^2^ energy input per switching, WeaVE provides 4.9°C expansion of the thermal comfort zone, which is equivalent to a continuous power input of 33.9 W/m^2^. This nonvolatile characteristic substantially decreases the required energy while maintaining the on-demand controllability, thereby providing vast opportunities for the next generation of smart personal thermal managing fabrics and wearable technologies.

**Significance Statement**

Personal thermoregulation offers tunable and localized thermal comfort for individuals. However, energy efficiency and wearability are often sacrificed for active controllability in these applications. We demonstrated the ultraefficient wearable variable-emittance (WeaVE) device to address the trade-off by modulating the heat transfer coefficient and introducing kirigami patterns. In contrast to the strategies of supplying heat/cooling power, this device can stay in the desired thermal comfort state without energy input, but it is still able to respond to the ambient temperature change by applying a small electrical potential. The incorporation of WeaVE and control circuits is also realized as a new scheme of autonomous tuning personal thermoregulation system.

## Introduction

Clothing has been one of the most essential inventions for tens of thousands of years. Because humans need to maintain thermal homeostasis, proper clothing can allow the human body to maintain the balance between body heat loss and metabolic heat generation, which is critical for productivity, health, or even survival. For example, under a cold environment, blood vessels contract to reduce the heat loss (vasoconstriction), redistributing blood back to the head and torso so that the vital organs can maintain the temperature for proper physiological functioning. However, this involuntary response to cold temperature also increases the blood pressure (1), triggers platelet aggregation (2), and inflammatory markers (3), which may increase the risks of thrombosis and cardiovascular diseases such as heart disease and ischemic stroke (4). The association between the extent of temperature change and the risk of stroke has been studied. Research shows the odds of stroke can be increased by 5–37% with a 5°F (2.78°C) additional environmental temperature variation (5, 6). This seasonal correlation can also be found in the human immune system (6), leading to profound effects on health.

For the same reason of maintaining thermal comfort, space heating and cooling technology also have a great influence on our lifestyle. However, the large mass and area of buildings require a huge amount of energy to reach thermal equilibrium and maintain the temperature gradient between indoor and outdoor. Buildings are responsible for about 40% of the energy usage and carbon dioxide emission in the United States and an annual energy bill of more than $400 billion. Among these consumptions, more than 40% of energy in households and business buildings are used for water and space heating, ventilation, and air conditioning (HVAC) purposes (7–9). In addition, another disadvantage of space temperature control is the lack of personalization. A smart garment allowing users to locally control the heat balance for personal health while reducing the HVAC energy consumption is in urgent need (1). Also known as personal thermal management, the wearable heating/cooling is a promising concept of reducing HVAC demand while achieving personalized thermal comfort and health (5, 6, 10–27).

The core concept of radiative tuning is the regulation of the overall emissivity, which is highly correlated to the extent of metallization of the textile. A highly metallic surface has a low emissivity, while a nonmetallic one can achieve radiative cooling by utilizing either the intrinsic large emissivity or the combination of the transmission and highly emissive human skin.

For instance, since the first framework of personal radiative cooling was proposed by adopting the concept of infrared-transparent visible-opaque fabric (ITVOF), nanoporous polyethylene (nano-PE) has been extensively used for radiative cooling textile (23, 24, 27, 28). As a nonmetallic material, nano-PE features the low infrared (IR) absorption due to the simple backbone structure and the high visible light scattering caused by the porous morphology. For the outdoor scenario, textiles with high reflectance in the solar spectrum are also developed to reduce the unfavorable absorption by introducing metal oxides, metafabric structures, and quantum dots (18, 23, 29, 30). On the other hand, by applying metal nanowire coating, the metallized nano-PE has a low emissivity and could achieve a passive heating performance (26). Low-emissivity textiles are usually based on metal nanowires to utilize their easy fabrication and brilliant stretchability (21, 25).

These studies above have achieved great performance in single-mode heating or cooling but do not have the responsiveness to ambient temperature change. Therefore, researchers tried to develop active materials that can modulate emissivity through morphology change, mechanical actuation, and thermoelectric devices (10, 16, 31, 32). A detailed comparison is made between this work and other personal thermoregulation devices in Tables S1–S3.

Nevertheless, personal thermal management still had the harsh trade-off between thermoregulation performance and power consumption as a wearable device. The traditional passive thermoregulation cannot be coupled with health informatics and wearable electronics, whereas the active thermoregulation (e.g. electric heater, recirculating water, and thermoelectric) is still energy intensive and bulky for long-term usage.

**Fig. 1.**
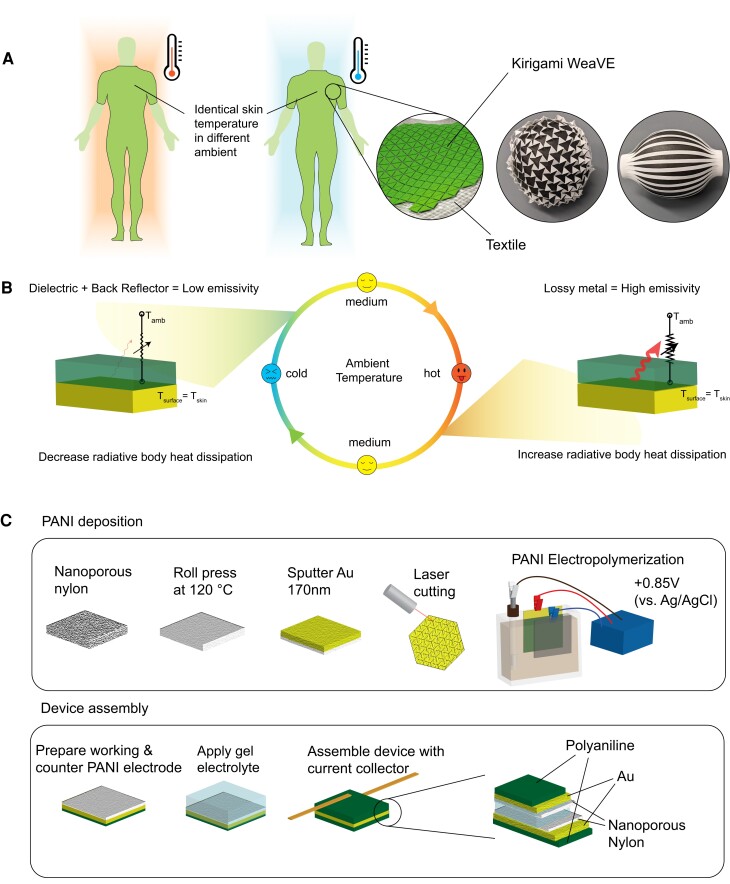
Kirigami-enabled WeaVE thermoregulator design and fabrication. A) Schematic of kirigami WeaVE. The kirigami cut allows the device to stretch and deform conformally along with the human body contour and movement. B) The working principle of emissivity tuning is based on the potential dependence of attenuation coefficient (imaginary part of refractive index) of PANI. C) PANI deposition and device assembly procedure.

In this paper, we developed a kirigami-enabled wearable variable-emittance (WeaVE) device that can use only a marginal energy input to electrochemically tune the radiative heat transport to help users maintain thermal comfort against ambient temperature fluctuation (Fig. 1A). The WeaVE device's thermoregulation is “adaptive,” which modulates the heat transfer coefficient rather than actively generating/pumping thermal energy. As a result, the energy consumption to maintain the heat balance is virtually zero, making it orders of magnitude more energy-efficient than its active counterparts. Furthermore, the kirigami pattern renders WeaVE devices stretchability and conformal deformability that can move along with the complicated morphology of the human body surface while fully preserving the electrochemical cell connection and configuration (33).

## Results

### WeaVE design and working principle

Among all types of heat transfer pathways, radiation stands out as the ideal mechanism for wearable thermoregulation devices for several reasons. First, the tunable range is significant. Reports have shown radiative heat transfer accounts for 45–50% of human body heat loss in an indoor scenario (34). Calculation based on ASHRAE Standard 55 also indicates that ∼33% of heat loss is through thermal radiation. Second, the active material for tuning can be ultrathin and lightweight, which are particularly critical for wearable applications. This can be explained by the simplified Stefan–Boltzmann law: ${\dot{q}}_{\mathrm{rad}}=\;e_{\mathrm{surf}}\sigma_{\mathrm{SB}}(T_{\mathrm{surf}}^{4}-T_{\mathrm{amb}}^{4})$, where ${\dot{q}}_{\mathrm{rad}}$ is the radiative heat flux, *e*_surf_ is the surface emissivity ranging between zero and unity, $\sigma_{\mathrm{SB}}$ is Stefan–Boltzmann constant, *T*_surf_ is the surface temperature, and *T*_amb_ is the ambient temperature. Note that we assume the ambient area is much larger and completely enclosing the object so that the view factor is close to unity and the influence of ambient emissivity is neglected. According to Kirchhoff's law, emissivity (*e*) is equal to absorptivity (*A*) at thermodynamic equilibrium. The emissivity (absorptivity) is determined by the attenuation length, which can be as thin as microns or submicrons for mid-IR thermal radiation. For opaque objects, the transmittance (*T*) is zero, so e=A=1−R−T=1−R, meaning that tuning reflectance (*R*) is the equivalent of tuning the emissivity and the radiative heat transfer.

As shown in Fig. 1B, our WeaVE device is a layered semisolid electrochemical cell focusing on mid-IR electrochromism. The device consists of a working/counter electrode pair, and both are electrodeposited polyaniline (PANI) on an Au-sputtered nanoporous nylon membrane. In recent years, PANI and other conjugated polymers, such as PEDOT:PSS, have been viewed as promising materials for high-range nonvolatile tunable optics (35–41). Prior research in electrochromic applications of PANI-based material focused on spacecraft thermal control rather than personal thermal management (42–53). In the working electrode, the underlying Au layer acts as a high-reflectivity (low-emissivity) layer. The PANI above will be electrochemically switched between a transmissive dielectric and a lossy metallic state, thereby realizing the mid-IR electrochromism that can vary emissivity to stabilize the radiative heat loss at a varying ambient temperature.

The optoelectrical property of PANI can be varied by switching continuously and reversibly between oxidation states. In acidic media, the three oxidation states of doped PANI are leucoemeraldine, emeraldine salt, and pernigraniline. Leucoemeraldine and emeraldine salt are the two favorable states in this device. The high transmittance of leucoemeraldine in mid-IR guarantees the low emissivity provided by the Au back reflector. On the other hand, when PANI is oxidized to emeraldine salt, its conductivity and subskin-depth thickness make it lossy and absorptive, therefore enhancing the emissivity of the device. The thickness of PANI was controlled by setting a specific polymerization charge during electrochemical deposition. The amount of PANI on the counter electrode is three times more than the working electrode, acting as a charge reservoir to ensure complete charge transfer and stable switching voltage. Ionic conductivity is provided by the poly(vinyl alcohol) (PVA)/poly(ethylene glycol) (PEG)-based gel electrolyte with sulfuric acid, which permeates in the Au-coated nanoporous nylon membrane. Prior to electropolymerization, kirigami patterns are defined by laser cutting, which is fast, precise, and scalable. In our earlier attempts, the laser-cut electrochemical cells were vulnerable to catastrophic crack propagation during stretching. Strong mechanical properties were accomplished by choosing nylon among other kinds of nanoporous membranes and implementing hot roll pressing (Fig. 1C).

### Mid-IR electrochromic property characterization

**Fig. 2.**
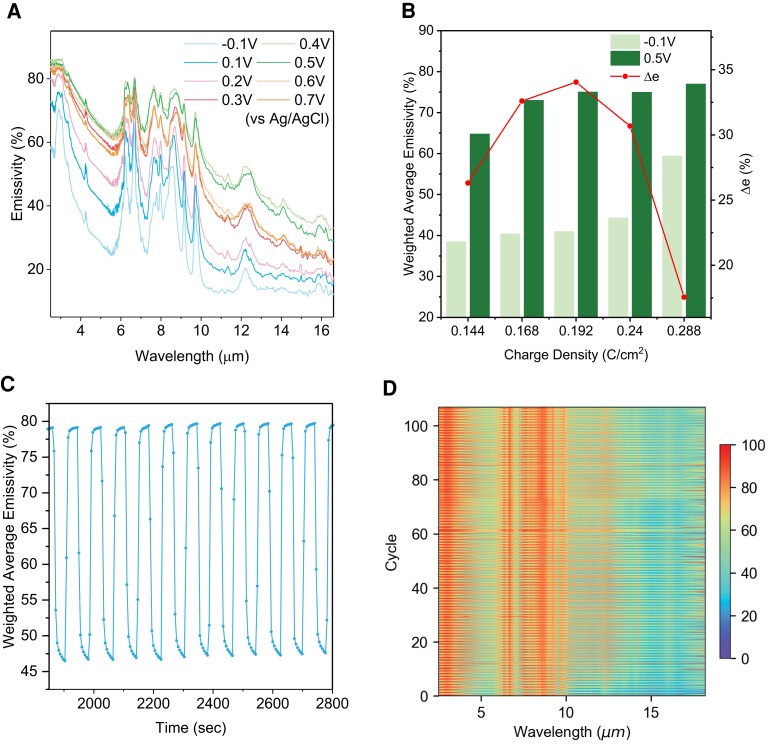
Dynamic mid-IR optical property characterization. A) Emissivity spectra of PANI/Au/nylon as a function of electrochemical potential. Note that the emissivity changes nonmonotonically, which reaches the maximum at +0.4 V and minimum at −0.1 V (versus Ag/AgCl). B) Emissivity and emissivity contrast dependence on PANI thickness (electrodeposited areal capacity). Thin PANI results in low emissivity in both states because the Au layer dominates. Thick PANI has too strong intrinsic absorption from both states due to molecular vibrational resonance. C) Representative cyclic emissivity spectra. Each data point represents the measured weighted-average emissivity based on black body radiation at 34°C. D) Time series of cyclic emissivity spectra.

As shown in Fig. 2A, PANI is electrochemically biased between −0.1 and 0.7 V versus Ag/AgCl, and its reflectivity was measured by the Fourier transform infrared (FTIR) spectrometer equipped with a diffuse gold integrating sphere. As there is no transmission in the presence of the Au layer, emissivity is calculated as 1-reflectance for each wavelength. At −0.1 V, the PANI is in leucoemeraldine form, which is more IR transparent, revealing the low emissivity of underlying Au, with an emissivity of ∼0.38. As the potential increases, the active layer is oxidized to the emeraldine salt form, enhancing the optical conductivity and emissivity simultaneously. At 0.4 V, the emissivity of PANI/Au reaches the maximum (∼0.65), which indicates the transformation into emeraldine salt is finished. The emissivity starts to decrease when applying a voltage larger than 0.4 V. The decrease of emissivity is mainly due to the presence of insulating pernigraniline, which is unfavorable because of the irreversibility and instability. The low conductivity of pernigraniline makes it more difficult for switching. Note that the thickness of the PANI-based conductive polymer lies in the subskin depth region, and the enhancement of emissivity is attributed to the increase in conductivity. Cyclic voltammetry, scanning electron microscopy, and Raman spectroscopy are also conducted to further understand the morphology and oxidation/reduction behavior of PANI (Figs. S1–S3).

Because of the small thickness of PANI, emissivity is determined by both PANI and the underlying Au layer together. At mid-IR frequency, Au is very close to a perfect electric conductor (PEC), but the thickness of PANI needs to be optimized to achieve large emissivity contrast, Δ*e*. As shown in Fig. 2B, samples with different thicknesses were prepared by varying the deposition areal density. Their emissivity spectra at −0.1 and +0.5 V (versus Ag/AgCl) were measured and weighted-averaged based on black body radiation at 34°C, the typical human skin temperature. The trade-off behavior between a large absorption coefficient and mid-IR transparency can be observed. Low electropolymerization charge density (0.144 C/cm^2^) provides higher reflection from the gold, but the thickness is not enough to provide high emissivity (absorptivity). On the other hand, when increasing thickness, both states are too dense and lossy, reducing the overall performance of the device. Consequently, Δ*e* is maximized at an intermediate value of 0.192 C/cm^2^.

The in situ emissivity tuning kinetics and cycle life measurement of WeaVE is conducted. In Fig. 2C, the representative cycle data show its fast switching and stability as an electrochromic layer. We can see PANI can reach 90% of the dynamic range within 16 s. Figure 2D shows the in situ cyclic spectra measurement. Each voltage is applied for 45 s (90 s per cycle). The experiment results showed a five percentage decrease in Δ*e* after 100 cycles of switching. The decrease in performance may result from the drying of the electrolyte or the drifting of equilibrium electrochemical redox potential of PANI. The drying issue can be solved by adding a passivation layer on the surface of the device, and the voltage drifting can be avoided by preconditioning the materials before assembly.

### Skin temperature stabilization

**Fig. 3.**
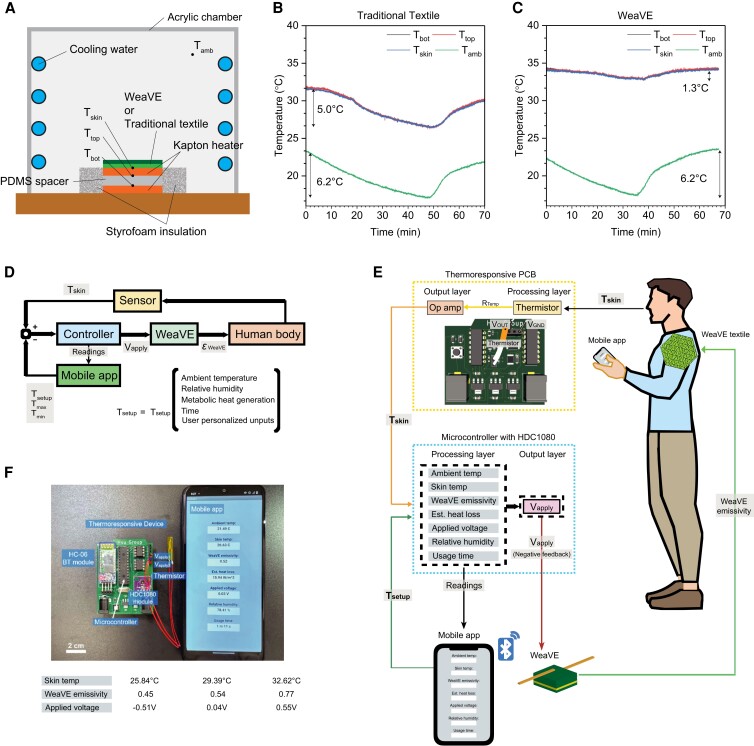
Heat transfer measurement and demonstration of artificial skin temperature stabilization. A) Schematic illustration of the heat transfer measurement chamber setup (not to scale) and the skin temperature under B) traditional textile and C) WeaVE device in a varying temperature environment. By adjusting the voltage applied to WeaVE, the artificial skin temperature was maintained within 1.3°C while the ambient temperature fluctuated by 6.2°C. D) The control loop of the autonomous personal thermoregulation system composed of WeaVE, controller IC, and wearable sensors. E) The schematic diagram that portrays the usage of WeaVE device for autonomous personal thermoregulation and temperature monitoring. F) Image of the personal thermoregulation system consisting of thermoresponsive unit, digital humidity and temperature sensor (HDC1080 module), Bluetooth module (HC-06), and the smartphone app.

To experimentally quantify the adaptability of the WeaVE device, we first demonstrate the ability of active tuning of the WeaVE device by placing it under a controlled varying temperature environment (Fig. 3A). WeaVE or traditional textile (58% cotton, 37% polyester, and 5% spandex) is attached on the top surface of a heating unit, which supplies a constant heat flux upward. The heating unit consisted of two stacked polyimide heaters with the same temperature, separated by a polydimethylsiloxane (PDMS) spacer. To ensure the heat flow is 1D and upward, a bottom auxiliary heater that follows the variation of the top heater with proportional–integral–derivative control is used. The temperatures of skin, ambient, top heater, and bottom heater, denoted as *T*_skin_, *T*_amb_, *T*_top_, and *T*_bot_, are measured and monitored by thermocouples. When *T*_top_ = *T*_bot_, there is no heat flow within the PDMS, and all the power supplied to the top heater will be upward, which is 105.2 W/m^2^ throughout the experiment.

The thermoregulation of WeaVE is demonstrated in Fig. 3C by comparing with traditional textile in Fig. 3B. Both traditional textile and WeaVE device are exposed to the same ambient temperature condition. When covered with traditional textile, the temporal variation in *T*_skin_ temperature has the same trend as the controlled ambient temperature, except for the lag in time due to the thermal inertia, which can be observed by the ∼7-min difference in the occurrence of the minimum temperature. With a 6.2°C transient variation, ranging from 23.3 to 17.1°C, traditional textile shows a 5.0°C temperature difference. On the other hand, WeaVE can stabilize the artificial skin temperature by adjusting the emissivity states at different voltages: begin with +0.7 V in the cooling state, and the voltage was decreased stepwise (0.1 V interval) to −0.7 V in heating state, and back to 0.7 V in the end. The time for applying different voltages is manually determined by observing the amount of *T*_skin_ decrease when conducting the experiment. By adjusting the emissivity and thus the radiative heat loss, WeaVE can reduce the artificial skin temperature fluctuation to 1.3°C while the ambient temperature fluctuates by 6.2°C. This means the WeaVE can expand the thermal adaptability by 6.2–1.3 = 4.9°C (8.8°F). To put this number in a daily life perspective, this 4.9°C (8.8°F) of expansion means the user will feel the same thermal comfort no matter the ambient temperature is 22.0°C (71.6°F) or 17.1°C (62.8°F).

Aside from thermoregulation, the heat transfer and electrochemical measurement also demonstrate the energy efficiency of WeaVE. In contrast with traditional HVAC systems that actively provide work input, WeaVE modulates the skin temperature by changing the emissivity, the intrinsic material property of the electrochromic layer. It is important to point out that WeaVE requires no energy to maintain its emissivity. We can calculate the effective power provided by WeaVE by comparing the heat transfer coefficient. The heat transfer coefficients in the low-*e* and high-*e* states are 8.98 and 6.79 W/m^2^K. That is, when the ambient and skin temperature difference is 15.9 K, the effective power is equivalent to $(8.98-6.79)\;W/m^{2}K\times 15.9\;K=34.8\;W/m^{2}.$ To put this energy consumption in the real-life perspective, if using an electric heater to supply the same heating power for the area of 1 m^2^, this electric heater will drain a flagship smartphone battery (4,500 mAh, 3.7 V) in less than 30 min. In contrast, WeaVE can indefinitely provide the warming effect with zero energy input because it is the heat transfer coefficient that is being tuned. We further include the energy for switching by considering the time series of current density and voltage of the redox reaction. As shown in Fig. S4, the switching energy per cycle is ∼5.58 mJ/cm^2^, which means a 1 m^2^ large WeaVE can switch more than 1,000 times by the aforementioned smartphone battery. Even if we conservatively assume the WeaVE is switched five times a day, it can still last for 200 days or several months of usage. This calculation clearly shows that WeaVE is orders of magnitude more efficient than traditional active thermoregulation due to its nonvolatile and reversible tuning mechanism.

To realize the functionality of autonomous tuning, we incorporate the sensing and controlling electronic components with WeaVE, including a thermistor, a humidity and temperature sensor (HDC1080), a Bluetooth module (HC-06), and smartphone application (Figs. S6 and S7). The control loop diagram is shown in Fig. 3D. The mobile app contains the user interface items, including the input of control parameters and the information of current system status (ambient and skin temperature, WeaVE emissivity, estimated heat loss, applied voltage, and relative humidity). Desired upper and lower limits of temperature tolerances are entered into the smartphone app and transmitted to the Arduino controller via Bluetooth. Skin temperature is detected by the thermistor and transmitted into the thermoresponsive unit (Fig. S8), and then emissivity and applied voltage to WeaVE are calculated. Ambient temperature and humidity, which are used to calculate the heat loss, are measured by HDC1080. The working principle illustration and the picture of the system are shown in Fig. 3E and F, respectively. The whole system is powered by lithium batteries. The photograph of the personal thermoregulation system is shown in the inset of Fig. 3F, where the upper and lower limits are at 23.0°C and 33.0°C, with ambient temperature at 22°C. The applied voltage is varied linearly within the temperature limits.

The heterogeneous deformation and the complex curved configuration on various parts of the human body result in the trade-off between the large contact area (device–human skin interface) and conformability, especially during deformation. Therefore, kirigami patterns are utilized to fit various body parts of humans. Here, different from existing wearable devices, we harness the correlation between the morphology (the principal curvature) of human skin and the maximum tensile strain in the attached device to guide the general design of the scalable and personalizable WeaVE device with zero-energy-cost emissivity adaptivity coupled with conformable capability.

**Fig. 4.**
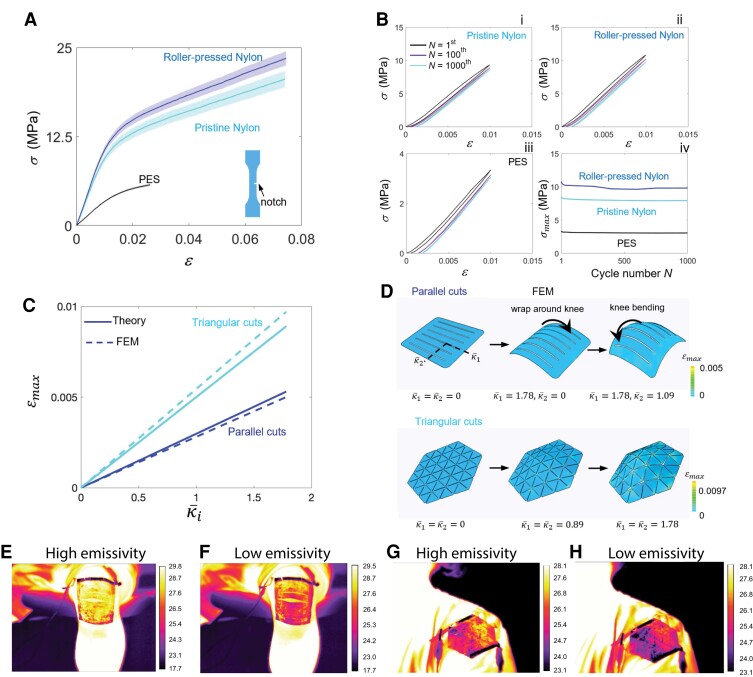
Kirigami WeaVE mechanical property study and thermal imaging. A) Tensile stress–strain curves of the notched pristine nylon, roller-pressed nylon, and PES sheets, each stretched to rupture. The nominal stress, σ, is defined as the force applied on the sheet, divided by the cross-sectional area of the undeformed specimen. B) (i–iii) Stress–strain curves of the notched pristine nylon, roller-pressed nylon, and PES sheets over cycles. (iv) The maximum stress for the three kinds of sheets as a function of the number of cycles. C) Maximum tensile strain $\varepsilon_{\max}$ as a function of the normalized principal curvature ${\bar{\kappa }}_{1}$. D) Simulation of the deformation of the (i) parallel and (ii) triangular precursors. The color bar represents the maximum tensile strain. ${\bar{\kappa }}_{1}\;$ and ${\bar{\kappa }}_{2}$ are the principal curvatures. E–H) Large-scale kirigami WeaVE thermal imaging on the knee E, F) and shoulder G, H). The voltage crossed the device electrodes are +0.5 V E, G) and −0.5 V F, H) for high-*e* (cooling) and low-*e* (heating), respectively.

To examine the mechanical stability and durability of the WeaVE device, we have conducted both the uniaxial tensile test (Fig. 4A) and the cyclic test of three different types of samples (Fig. 4B) with an introduced small notch (inset of Fig. 4A), including roller-pressed nylon (the materials used for the WeaVE device) and pristine nylon and polyethersulfone (PES) for comparison. Figure 4A shows the stress–strain curves of the three samples. Compared with PES, both nylon samples show a much higher fracture strain and stiffness, thus leading to a higher toughness. The toughness of the nylon and roll-pressed nylon are measured to be 85.69 kJ/m^2^ and 97.34 kJ/m^2^, respectively (Fig. S9), indicating that the toughness of the nylon sheet is enhanced by roll pressing (Fig. 4A). Moreover, the low cycle fatigue tests under strain control of the three notched sheets show that all three types of samples exhibit negligible hysteresis and permanent deformation, as shown in Fig. 4B. After 1,000 cycles of loading and unloading, the residual strain is about 0.1% (Fig. 4B, i–iii), and the maximum stress of each curve almost does not change (Fig. 4B, iv). The stability and notch insensitivity of the device support the application of kirigami designs to improve conformability (Figs. S10 and S11).

Here, we choose two different patterning cuts, triangular and parallel, to make the device conformable to different parts of the human body that undergo different levels of bending strains (Fig. 4C and D). As validated by both the finite element method (FEM) simulation (Fig. 4D) and the experiments, the WeaVE device deforms with the human skin conformably. The parallel cuts facilitate bending for directions both along and perpendicular to the cuts and make each ribbon a geodesic curve of the human skin, where conformability and adaptivity arise naturally. Thus, the parallel cut pattern is especially suitable for joints such as knees and elbows. As shown in Fig. 4D, i, the parallel cut precursor is first bent to fit and wrap around the curved configuration of the human knee, where the normalized bending curvature ${\bar{\kappa }}_{1}$ increases from 0 to 1.78 (i.e. the curvature of the knee in direction 1). Then when the knee bends, it bends with the knee smoothly with the bending curvature ${\bar{\kappa }}_{2}$ increasing from 0 to 1.09 (the curvature of the knee in direction 2 when the bending angle is 90°). ${\bar{\kappa }}_{1}$ and ${\bar{\kappa }}_{2}$ are the principal curvatures normalized by the width of the human knee. On the other hand, the triangular cut pattern exhibits an approximate isotropic morphology feature, and its morphology varies smoothly with the inhomogeneous deformation of the human skin (Fig. 4D, ii). When stretched by the human skin, the triangular network opens progressively to fit the skin with both ${\bar{\kappa }}_{1}$ and ${\bar{\kappa }}_{2}$ increasing from 0 to 1.78.

Further, to make our device scalable and personalizable, a general and scale-independent correlation between the normalized principal curvature ${\bar{\kappa }}_{i}$ and the maximum principal tensile strain is derived both theoretically and numerically. As shown in Fig. 4C, the maximum principal tensile strain $\varepsilon_{\max}$ in the device increases linearly with increasing curvature ${\bar{\kappa }}_{i}$ of the human skin. It is also noteworthy that the maximum tensile strain of both patterns is much smaller than the fracture strain (Fig. 4B), which shows the stability and robustness of the WeaVE device.

In the fabrication process of kirigami WeaVE, a SEBS passivation layer was coated on the surface to prevent electrolyte overflow and drying. In Fig. 4E–H, the thermal image of two volunteers wearing kirigami WeaVE was captured in an ambient temperature of 17°C at 0.5 V and −0.5 V, respectively. The image visualizes the “apparent temperature” change when WeaVE device is in both states. Note that lower apparent temperature represents smaller amount of thermal radiation reaching the IR camera, which corresponds to low-*e* state (Fig. 4F and H), and vice versa. For a small temperature change, the apparent temperature change can be approximated as linearly related to the thermal radiation power, which is also equivalent to the level of thermoregulation (Fig. 3C). Compared with bare skin, the area covered with WeaVE has a tunability of apparent temperature of ∼2.5°C, representing the adjustable thermal insulation. Moreover, because the mid-IR wavelength range greatly differs from visible light, we can apply a visibly colored, mid-IR transparent layer to render the WeaVE device with different visual appearance for aesthetic preference without affecting the radiative thermoregulation performance (Fig. S5).

## Conclusion

In conclusion, WeaVE demonstrates its energy efficiency, adaptive emissivity modulation, and wearability. By incorporating electrochromism and kirigami, WeaVE has the advantages of control and personalization like active devices but requiring zero energy when maintaining the heating/cooling state, which is an essential advantage for long-term wearable applications. The users can adapt and determine the expected temperature by simply applying a low voltage. A small, lightweight, portable battery or device will satisfy the need for week-long usage. Furthermore, WeaVE has the potential to be integrated with other types of wearable sensors, so that autonomous thermoregulation can be achieved by health-oriented decision loops based on multiple data inputs from the physiological, environmental, and psychological cues. As WeaVE exhibits the adaptability using electrochromism and versatility by kirigami, we envision exciting future research works in theory and application about mid-IR modulation, wearable thermal management devices, and solid mechanics. In parallel, WeaVE opens up the possibility in engineering by incorporating between wearable device systems, radiative thermal management, and textile engineering.

## Materials and methods

### Fabrication of WeaVE

First, the nylon substrate with pore sizes of 0.1 µm was roll-pressed at 120°C, and a layer of 170-nm-thick Au was sputtered. Commercially available nanoporous nylon membrane was purchased from Tisch. Next, PANI was electropolymerized at constant 0.85 V versus Ag/AgCl (3 m NaCl) in the electrolyte on the Au-sputtered nylon. The electrolyte is prepared by adding aniline and diphenylamine monomer (Sigma-Aldrich), in 0.21 m and 0.01 m, respectively, in 0.5 m sulfuric acid (Sigma-Aldrich) and 0.6 m dopant polyanetholesulfonic acid sodium salt (Sigma-Aldrich). The surface of the PANI electrode is then rinsed in water to remove undesired oligomers.

The solid electrolyte is a 0.5 m sulfuric acid solution containing 7 wt.% PVA (*M*_w_ 89,000–98,000) and 3 wt.% PEG (average *M*_n_ 600). The electrolyte is heated in oil bath to 90°C until PVA and PEG are fully dissolved. A thin layer of electrolyte is applied onto the back of the counter electrode for 10 min in a well-ventilated area for drying before combining with PANI-deposited working electrode. The current collector attached is a double-sided copper tape (3M).

### FTIR measurement

The mid-IR diffuse reflectance spectra of samples from 2.5 to 16.67 µm were measured by an FTIR spectrometer (model 6700, Thermo Fisher Scientific) accompanied by a diffuse gold integrating sphere (PIKE Technologies). Standard gold reference was chosen as the background of the measurement. Cyclability test is conducted in situ at ±0.6 V in two-electrode mode using the same FTIR for reflectance spectra measurement. A total of 107 cycles were performed.

### Real-time heat transfer measurement

The real-time heat transfer coefficient and skin temperature were measured by a power supply and a testing module. The structure of the measurement chamber is illustrated in Fig. 3A in the main text. The testing module consists of two heaters (top and bottom), a spacer, and thermocouples. Both heaters were flexible polyimide heater plates (1 inch × 1 inch, OMEGA Engineering). Two K-type thermocouples (SKA1, OMEGA Engineering) were attached to the center of the top and bottom surface in the testing heater. A PDMS spacer (1 inch × 1 inch, OMEGA Engineering) was sandwiched below the testing heater.

### Kirigami

The parallel cut kirigami WeaVE (10 cm × 10 cm square) was hand-cut by a hobby knife after finishing the device assembly process. The width of the stripe is 7 mm, and a ∼1-cm-thick space to each side of the device is reserved. The triangular cut kirigami WeaVE was laser-cut with a 60-W laser cutter before PANI electrodeposition. The device was a hexagon with a side length of 4.56 cm. The “Y-shaped” cuts consist of three 8-mm-long cuts, constructing 120° angles with each other.

### Tensile test

The uniaxial stretching and the cyclic test are performed using Instron 5944 with a 500-N load cell based on the ASTM methods. The strain rate is 1 mm/min. The 0.5 mm notch is processed by laser cutter (Epilog 40W).

The toughness is expressed as:


$$\Gamma =HW(\lambda_{C}),$$

where $W(\lambda_{C})$ is the energy density obtained by integrating the area below the stress–strain curve of the unnotched samples and $\lambda_{C}$ is the strain when the crack begins to propagate. *H* denotes the distance between the two grippers before deformation.

### FEM

In the FEM simulation (Abaqus/Standard), the nylon sheets corresponding to the parallel and triangular cuts are modeled as linear elastic, isotropic material with Young's modulus *E* of 400 MPa and Poisson's ratio *µ* of 0.3. The geometries are meshed with solid quadratic tetrahedral elements (C3D10H), and a fine mesh is applied to the connection area. For the triangular precursor, the boundary edges are fixed, and a uniform pressure is applied to the bottom face following the variation of the curvature of the human skin. The parallel precursor is first attached to fit the curved surface of the human knee and then is bent with the rotation of the human knee.

### Relationship between the strain and the principal curvature

For the parallel precursor, the maximum tensile strain $\varepsilon_{\max}$ generated by the bending of the deformed precursor is expressed as:


$$\varepsilon_{\max}=Ct\kappa /2,$$

where *t* = 0.5 mm and κ=0.0178 mm^−1^ denote the thickness of the sheet and the principal curvature, respectively. C=1.2 is the stress concentration factor.

For the triangular precursor, the maximum tensile strain $\varepsilon_{\max}$ results from the opening of the hinge and is expressed as:


$$\varepsilon_{\max}=Cw\Delta k/2,$$

where *w* and Δk denote the width of the hinge in the triangular precursor and the variation in the curvature of the hinge, respectively. *C* is the stress concentration factor. The Gaussian curvature of the structure is expressed as $K=\;-\frac{1}{2\lambda }\nabla^{2}(2\lambda )=3.19\times 10^{-4}\;(mm^{2})$, where λ is the swelling factor and $\nabla^{2}$ is the Laplace operator. Next, the applied strain $\varepsilon_{\mathrm{app}}\approx 0.071$ and the opening angle θ=0.085 are derived based on the variation of the metric tensor $g_{ij}$ that is in the form of $\Delta g_{ij}=2\varepsilon_{ij}=2\sqrt{1+\sqrt{3}\sin\;(\theta )+2\mathrm{si}n^{2}\left(\frac{\theta }{2}\right)}-2$. Then, we assume that the variation in the curvature of the hinge increases linearly with the opening angle of the triangular unit and is given by $\Delta k=\frac{0.3\theta }{\pi }$. Thus, the maximum tensile strain is expressed as $\varepsilon_{\max}=\frac{Cw\Delta k}{2}\approx 0.0089$. Also, the variation in the curvature of the hinge increases linearly with the principal curvature κ of the triangular cut. Note that the curvatures in Fig. 4 are normalized by the width of the human knee (100 mm).

## Supplementary Material

pgad165_Supplementary_Data

## Data Availability

All data are included in the manuscript and/or Supplementary material.
