# Correction to: A kirigami-enabled electrochromic wearable variable-emittance device for energy-efficient adaptive personal thermoregulation

**DOI:** 10.1093/pnasnexus/pgad378

**Published:** 2023-11-15

This is a correction to: Ting-Hsuan Chen, Yaoye Hong, Ching-Tai Fu, Ankita Nandi, Wanrong Xie, Jie Yin, Po-Chun Hsu, A kirigami-enabled electrochromic wearable variable-emittance device for energy-efficient adaptive personal thermoregulation, *PNAS Nexus*, Volume 2, Issue 6, June 2023, pgad165, https://doi.org/10.1093/pnasnexus/pgad165

During a retroactive audit conducted by *PNAS Nexus*, it was discovered that this paper was missing a statement acknowledging compliance with the *PNAS Nexus* Human and Animal Participants and Clinical Trials policy:

The “two volunteers” stated in our article means two members, Ting-Hsuan Chen and Ankita Nandi, in our research team, as stated in the author contribution section. Both research team members designed the experiment and captured the thermal images together. They understood that the action of capturing thermal camera images presents no risk of harm.

This error has been corrected in the original article.

