# Supplementary material for: A kirigami-enabled electrochromic wearable variable-emittance device for energy-efficient adaptive personal thermoregulation
Source: PNAS Nexus. 2023 Jun 13;2(6):pgad165. doi: 10.1093/pnasnexus/pgad165 (PMC10263260; doi:10.1093/pnasnexus/pgad165)
Supplement: pgad165_Supplementary_Data [file pgad165_supplementary_data.zip › PNASNEXUS-PNASNEXUS-2023-00269-T-s01.pdf]

Supplementary Information for

A Kirigami-enabled Electrochromic Wearable Variable Emittance (WeaVE) Device for Energy-Efficient Adaptive Personal Thermoregulation

Ting-Hsuan Chen[†1], Yaoye Hong[†2], Ching-Tai Fu[1,3], Ankita Nandi[1,4], Wanrong Xie[1,5], Jie Yin[2*], Po-Chun Hsu[1,6*]

[1]Thomas Lord Department of Mechanical Engineering and Materials Science, Duke University, Durham, North Carolina 27708, United States

[2]Department of Mechanical and Aerospace Engineering, North Carolina State University, Raleigh, NC 27695, USA

[3]Ming Hsieh Department of Electrical and Computer Engineering, University of Southern California, Los Angeles, California 90089, United States

[4]Department of Applied Physics and Materials Science, California Institute of Technology, Pasadena, CA 91125, USA

[5]Department of Applied Physical Sciences, University of North Carolina at Chapel Hill, Chapel Hill, NC, 27599, USA

[6]Pritzker School of Molecular Engineering, The University of Chicago, Chicago, IL 60637, USA

[†] These authors contributed equally to this work.

*Corresponding author email: jyin8@ncsu.edu, pochunhsu@uchicago.edu

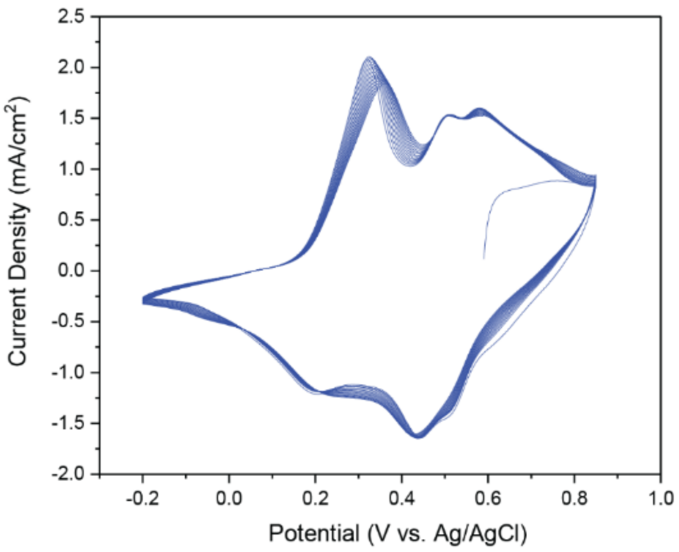

Fig. S1. Cyclic voltammogram of a WeaVE device electrode. The CV were scanned at 5 mV/s for 10 cycles continuously from -0.2 V to 0.85 V, using Ag/AgCl(3M NaCl) reference electrode. The anodic scan shows the presence of three oxidation peaks at 0.3V (leucoemeraldine, LE) to radical cationic polaron, 0.5V (oxidation of intermediate species) and 0.6V (polaronic to bipolaronic emeraldine salt, ES). The dark green color of ES is observed when the anodic scan is performed.

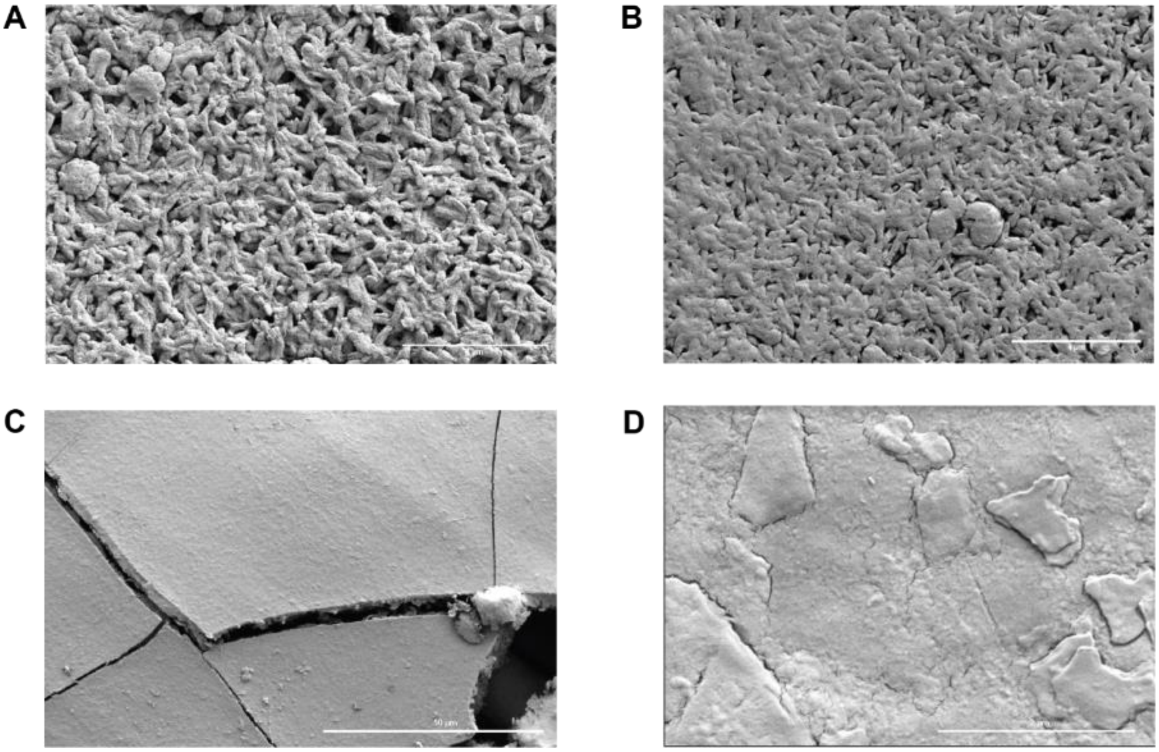

Fig. S2. SEM images of Au-sputtered (A) pristine and (B) roller-pressed nanoporous nylon substrate. Randomly distributed porous network structure with similar pore size can be observed on both samples, while the roller-pressed Au/nylon has a squeezed and smoother surface with smaller pores. (C-D) Morphology of electrodeposited PANI thin film on Au/nylon at different magnification under SEM. At a lower magnification, PANI film surface is compact, uniform and smooth. Minor cracks and roughness are presented on the film at the micrometer level.

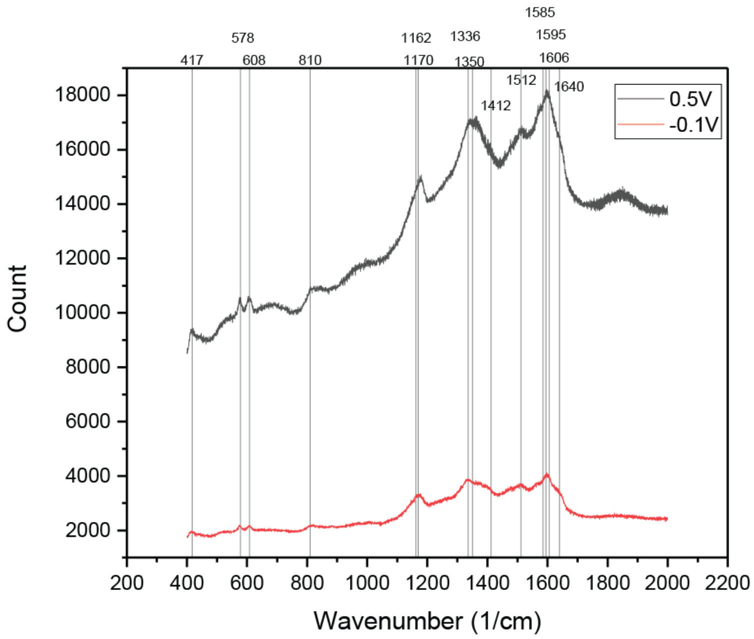

Fig. S3. Raman spectroscopy of emeraldine salt and leucoemeraldine. First, the peaks at 410, 578 and 608 correspond to the out-of-plane ring deformation, benzene ring deformation, and $\nu(C-S)/SO_2$, which is stronger in emeraldine salt due to its doping nature. The peaks at 810, 1162/1170, 1336/1350, 1512, and 1585/1595/1606 cm^{-1} are assigned to the out of plane C-H motions, in-plane bending of C-H in semi-quinoid units, C-N stretching, N–H bending in semiquinoid rings, and C=C stretching in the semiquinoid rings, respectively. Quinoid and semiquinoid ring peaks are also stronger in emeraldine salt, but their presence in leucoemeraldine form suggests the incomplete reduction in PANI.

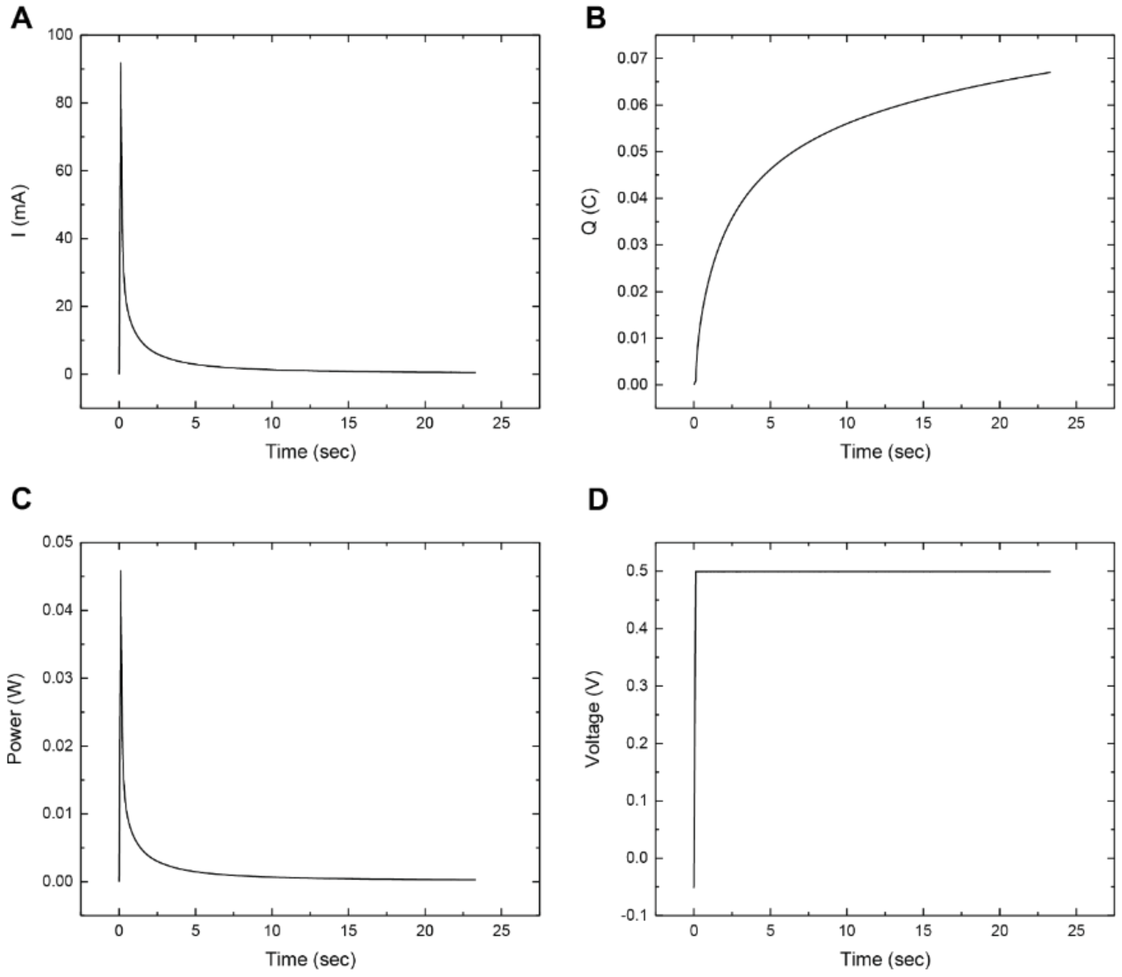

Fig. S4. Area energy density of WeaVE in a single switching process. (A) current, (B) charge (C) power and (D) voltage of working electrode vs. time. By multiplying the amount of charge and voltage applied, divided by the area of WeaVE, 6 cm^2, the area energy density is 0.067 C × 0.5 V / 6 cm^2 = 5.58 mJ/cm^2.

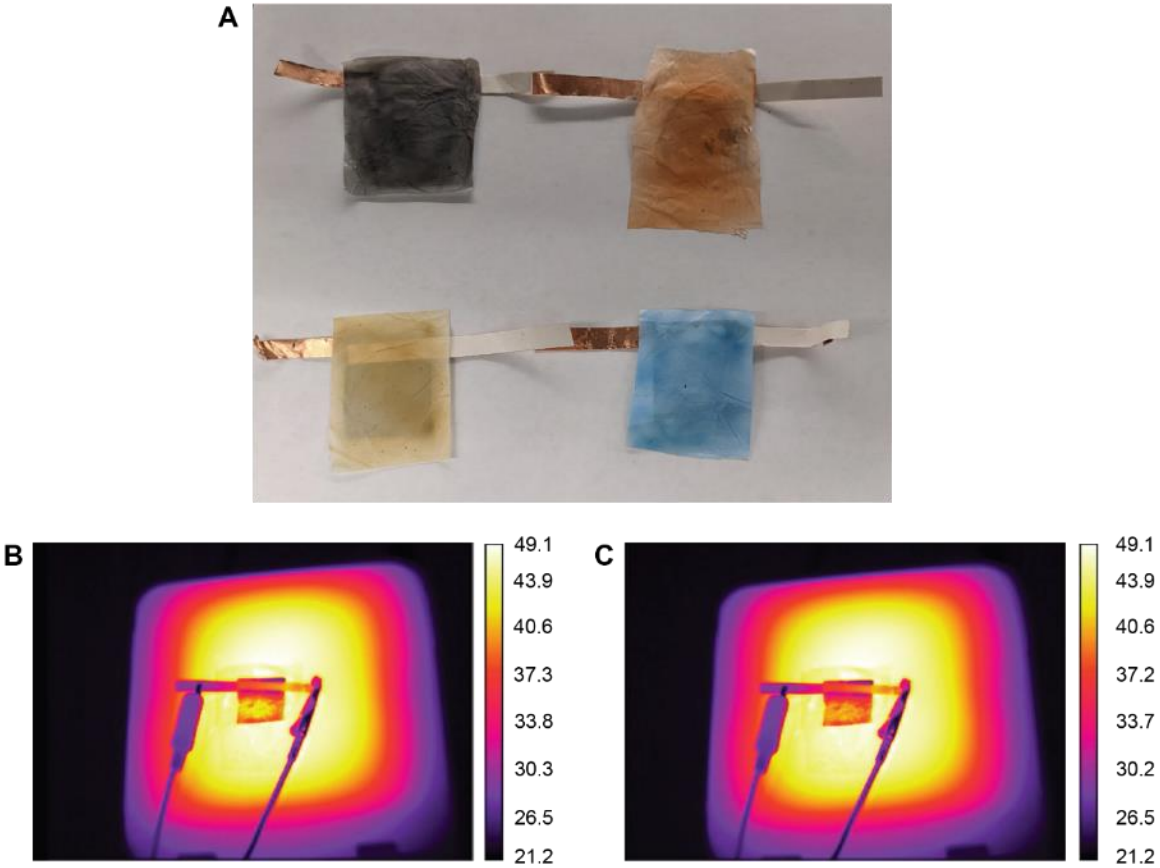

Fig. S5 (A) Optical image and thermal images of (B) high-*e* (cooling) and (C) low-*e* (heating) state of pigment-decorated WeaVE. This shows WeaVE is also functional with a colored IR-transmissive PE film covered on the top. The color is applied by spraying different nanoparticles on nanoporous PE.

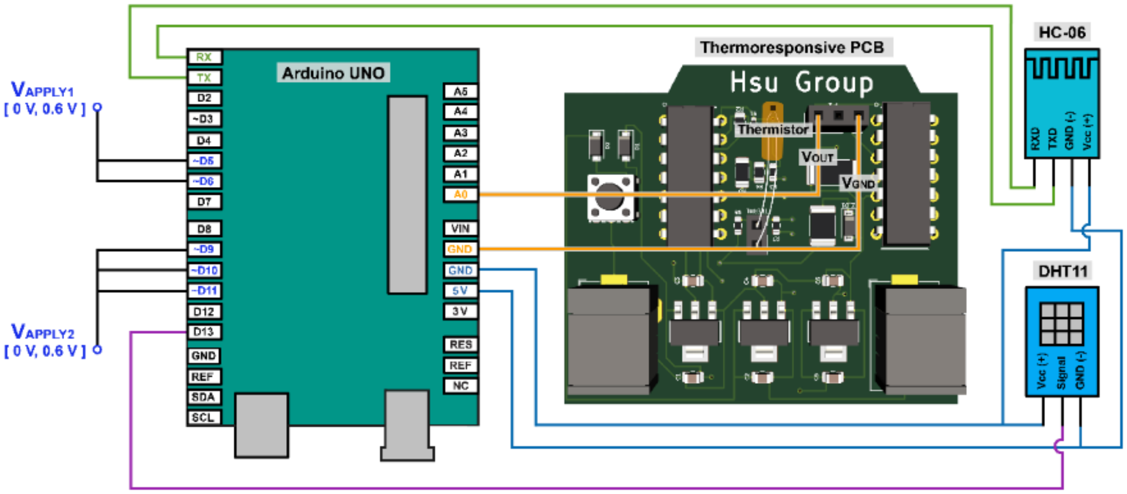

Fig. S6 Circuitry of the autonomous personal thermal management system. Arduino Uno, Thermoresponsive PCB, HC-06 Bluetooth module, and DHT11 are connected to construct the device. V_{out} and GND of the Thermoresponsive PCB are connected to A0 and GND pin of the Arduino Uno, respectively. HC-06 Bluetooth module's VCC (+5 V), GND, TXD, and RXD pins are connected to 5 V, GND, RX, and TX pin of Arduino Uno, respectively. VCC (+5 V), GND, and Signal pin of DHT11 are connected to 5 V, GND, and #13 Digital pin of the Arduino, respectively. Digital pins #5 and #6 are connected in parallel (represented by $V_{APPLY}1$), and, similarly, Digital pins #9, #10, and #11 are also connected in parallel (represented by VAPPLY2), both of which are then connected to WeaVE.

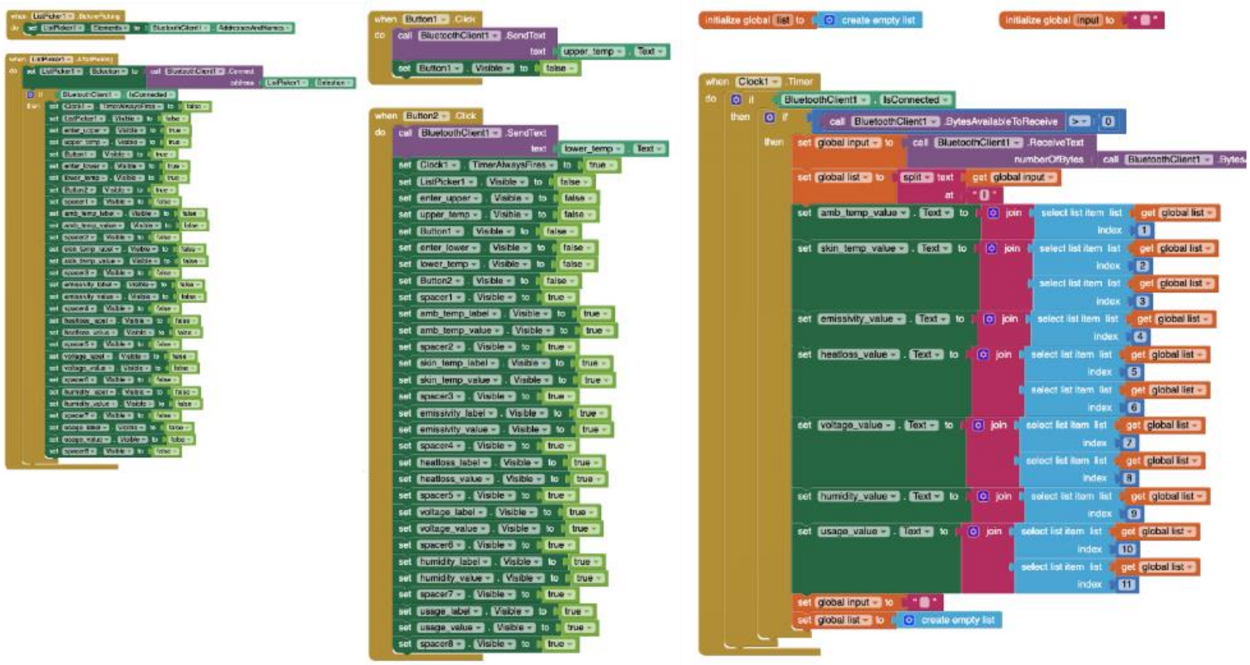

Fig. S7. Mobile App for the Autonomous Personal Thermal Management System. Placement of the MIT App Inventor logic blocks to function the mobile app. The mobile app consists of connecting to the Bluetooth client, transmitting the desired set points of upper and lower temperature tolerances to Arduino, and receiving thermal statuses of ambient temperature, skin temperature, WeaVE emissivity, estimated heat loss, applied voltage, relative humidity, and usage time that are displayed on the mobile app.

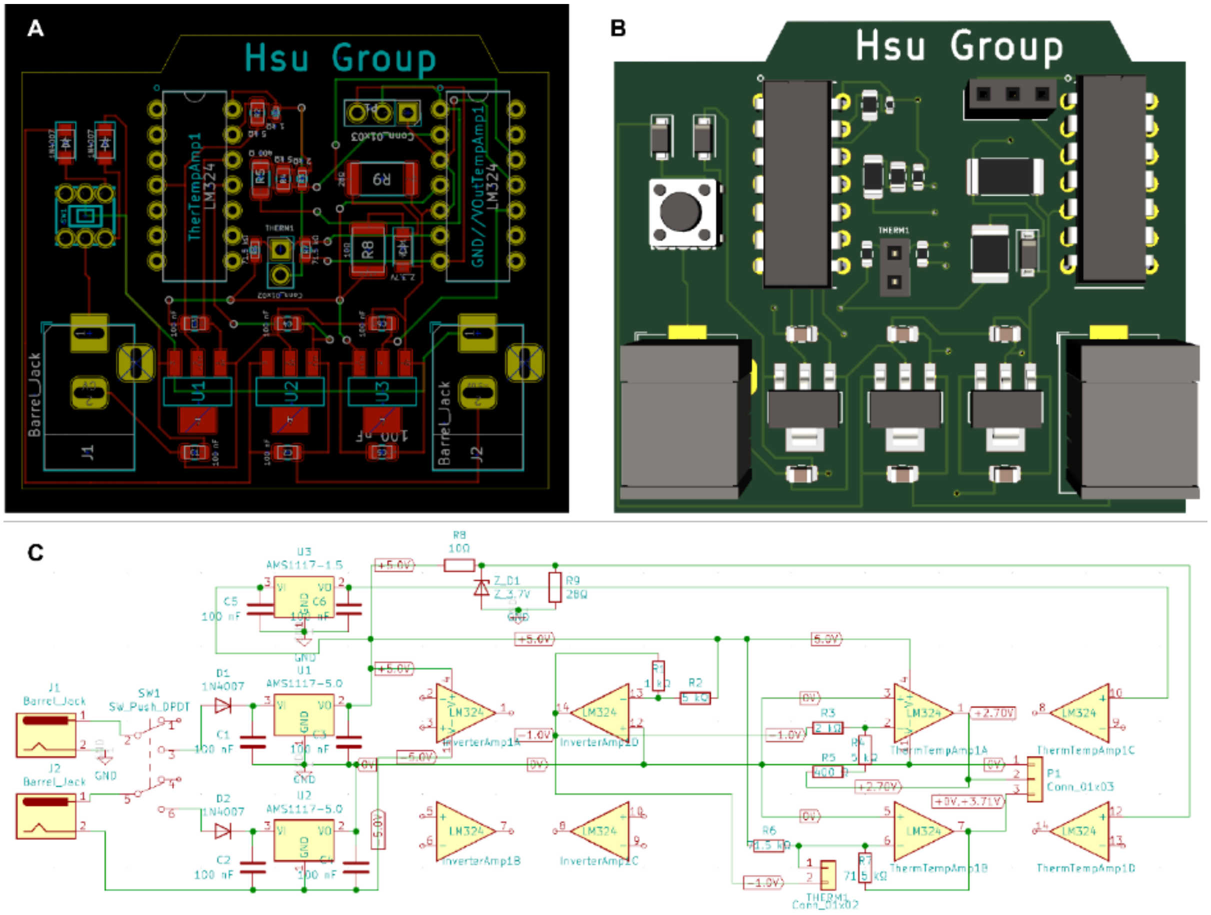

Fig. S8. (A) Printed circuit board layout, (B) 3D schematic with placed components, and (C) circuitry of the thermoresponsive PCB. The LM324 on the left serves an inverter that outputs a voltage of -1 V with one of its operational amplifiers. The -1 V then passes through the thermistor and enters the inverting input of an operational amplifier of the other LM324 to output a positive voltage. Detailed calculations regarding circuitry calculations are further discussed in Figure S3. Two AMS1117 voltage regulators of 5 V are powered by individual 9 V batteries, providing voltages of +/-5 V when connected in series. The 1.5 V AMS1117 voltage regulator is powered by the battery supplying the +5 V AMS1117 voltage regulator. A 3.7 V Zener diode is employed for a desired 3.7 V output. Similarly, one of the operational amplifiers is set to output 2.7 V. These discrete voltages of 3.7 V, 2.7 V, and 1.5 V are designed to increase functionality and can be soldered to other operational amplifiers if necessary. Designed Thermoresponsive PCB are manufactured by JLCPCB.

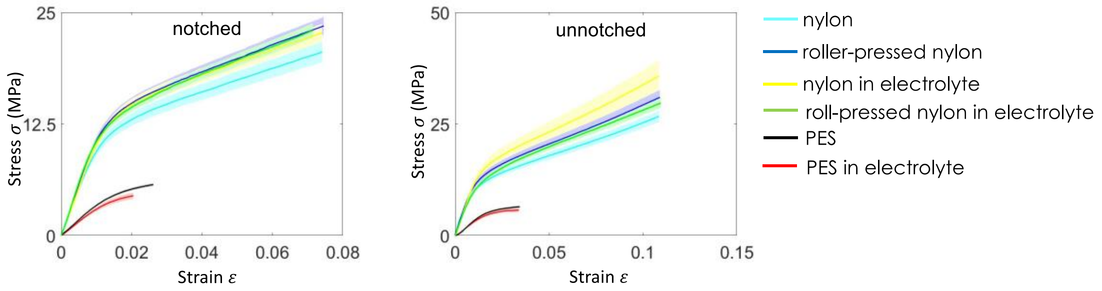

Fig. S9 Tensile stress-strain curves of the notched and unnotched sheets, each stretched to rupture. The nominal stress, σ, is defined as the force applied on the sheet, divided by the cross-sectional area of the undeformed specimen.

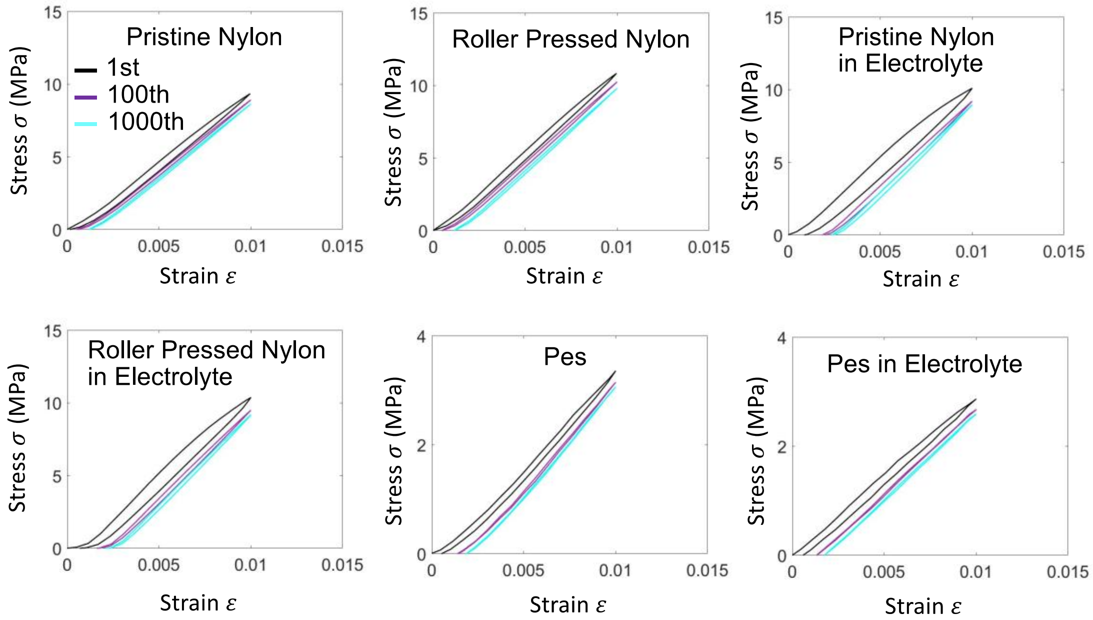

Fig. S10. The stress-stretch curves of the six types of notched sheets over cycles with ε = 0.01

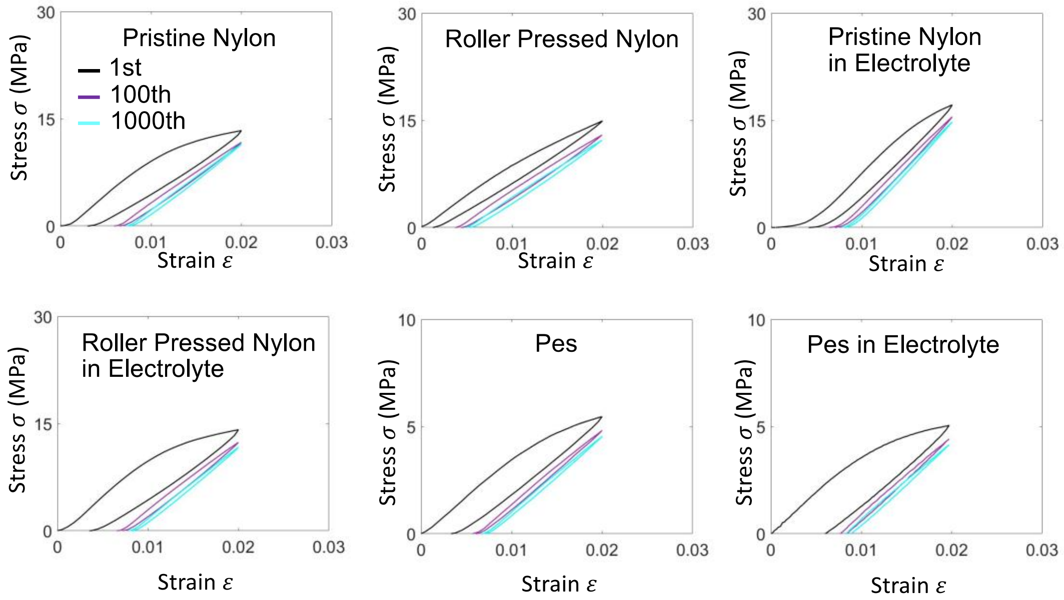

Fig. S11. The stress-stretch curves of the six types of notched sheets over cycles with ε = 0.02

Video S1. FEM simulation of parallel kirigami cut

Video S2. FEM simulation of triangular kirigami cut

Ref.	Strategy	Responsive to environment	Maintain thermal comfort state without energy input	Actively controllable	Kirigami design
[1]	Moisture responsive IR-gating	V	V		
[2-3]	Thermoelectric heater/cooler	V		V	
[4-9]	NanoPE-based cooling		V		
[7,10-11]	Metallized fabric		V		
[13,14]	Dual-mode textile		V		
[10,14-16]	Joule heating			V	

[17]	Graphene intercalation	V		V	
[18]	Mechanical strain		V	V	
[19-20]	Aerogel insulation		V		
[21]	Radiative cooling by metafabric		V		
This work	Electrochromic conductive polymer	V	V	V	V

Table S1. Strategies of personal thermoregulation

	Mechanism of shape-morphing	Stretching-to-bending	Maintain thermal comfort state without energy input	Thermal regulation performance	Principle/application
[22]	Stretching in connection joints		V	2.3°C single-mode cooling	Breathable nanofibrous/ personal thermoregulation
[23]	Rotation hinge			N/A	Electric armor
[24]	Stretching in connection joints			N/A	Sensor
[25]	Stretching in connection joints			$\frac{\lvert\Delta T\rvert}{\Delta\sigma} =$ 0.28 °C/MPa	Elastocaloric/ personal thermoregulation
[26]	stretching in connection joints			Joule heating	Joule heating/ personal thermoregulation
[27]	stretching in connection joints			$\Delta\varepsilon$ <0.20 4–15 um	Aerogel/thermal stealth
This work	Universally conformable	V	V	$\Delta\varepsilon$ =0.35 2.5-16.6 um, Thermal	Conductive polymer electrochromic/ personal thermoregulation

	capability design			comfort zone expansion 4.9°C	

Table S2. Comparison to other recent kirigami-enabled personal thermoregulation wearable devices.

	Responsive to environment	Maintain thermal comfort state without energy input	Actively controllable	Kirigami design	Emissivity regulation	Principle
[1]	V	V			$\Delta\varepsilon$ =0.35	Moisture responsive
[2]	V		V		Thermal comfort zone expansion 14°C	Thermoelectric
[17]	V		V		$\Delta\varepsilon$ = ~0.30<br>0.9-13um	Graphene intercalation
[12]		V			ε =0.8 (cooling) or 0.2(warming)	Dual-mode
[13]		V			ε =0.9 (cooling) or 0.3(warming)<br>2.5–18 um	Dual-mode
[18]		V	V		ΔT =0.40<br>4.5–16.5 um	Mechanical

[15]			V		Fixed skin temperature at 42°C	Joule heating+electronics
This work	V	V	V	V	$\Delta\varepsilon$ =0.35 2.5-16.6 um, Thermal comfort zone expansion 4.9°C	Conductive polymer electrochromic

Table S3. Performance comparison with other smart radiative textile.

References

1. Zhang, X. A., et al. (2019). "Dynamic gating of infrared radiation in a textile." Science 363(6427): 619-623.
2. Hong, S., et al. (2019). "Wearable thermoelectrics for personalized thermoregulation." Science Advances 5(5).
3. Zhang, T., et al. (2017). "High-performance, flexible, and ultralong crystalline thermoelectric fibers." Nano Energy 41: 35-42.
4. Hsu, P.-C., et al. (2016). "Radiative human body cooling by nanoporous polyethylene textile." Science 353(6303): 1019-1023.
5. Cai, L., et al. (2019). "Temperature Regulation in Colored Infrared-Transparent Polyethylene Textiles." Joule 3(6): 1478-1486.
6. Peng, Y., et al. (2018). "Nanoporous polyethylene microfibres for large-scale radiative cooling fabric." Nature Sustainability 1(2): 105-112.
7. Yang, A., et al. (2017). "Thermal Management in Nanofiber-Based Face Mask." Nano Lett 17(6): 3506-3510.
8. Cai, L., et al. (2019). "Temperature Regulation in Colored Infrared-Transparent Polyethylene Textiles." Joule 3(6): 1478-1486.
9. Cai, L., et al. (2018). "Spectrally Selective Nanocomposite Textile for Outdoor Personal Cooling." Advanced Materials 30(35): 1802152.
10. Hsu, P.-C., et al. (2015). "Personal Thermal Management by Metallic Nanowire-Coated Textile." Nano Letters 15(1): 365-371.
11. Cai, L., et al. (2017). "Warming up human body by nanoporous metallized polyethylene textile." Nature Communications 8(1): 496.
12. Gao, Q., et al. (2021). "Breathable and Flexible Dual-Sided Nonwovens with Adjustable Infrared Optical Performances for Smart Textile." Advanced Functional Materials 32(5).
13. Hsu, P.-C., et al. (2017). "A dual-mode textile for human body radiative heating and cooling." Science Advances **3**(11): e1700895.
14. Jo, H. S., et al. (2018). "Wearable transparent thermal sensors and heaters based on metal-plated fibers and nanowires." Nanoscale 10(42): 19825-19834.
15. Jang, J., et al. (2017). "Rapid production of large-area, transparent and stretchable electrodes using metal nanofibers as wirelessly operated wearable heaters." NPG Asia Materials 9(9): e432-e432.

16. Choi, S., et al. (2015). "Stretchable Heater Using Ligand-Exchanged Silver Nanowire Nanocomposite for Wearable Articular Thermotherapy." ACS Nano 9(6): 6626-6633.
17. Ergoktas, M. S., et al. (2020). "Graphene-Enabled Adaptive Infrared Textiles." Nano Letters 20(7): 5346-5352.
18. Leung, E. M., et al. (2019). "A dynamic thermoregulatory material inspired by squid skin." Nat Commun 10(1): 1947.
19. Jiang, Y., et al. (2017). "Preparation and characterization of thermal protective aluminum hydroxide aerogel/PSA fabric composites." Journal of Sol-Gel Science and Technology 82(2): 370-379.
20. Liu, Z., et al. (2019). "Nanofibrous Kevlar Aerogel Threads for Thermal Insulation in Harsh Environments." ACS Nano 13(5): 5703-5711.
21. Zeng, S., et al. (2021). "Hierarchical-morphology metafabric for scalable passive daytime radiative cooling." Science 373(6555): 692-696.
22. Li, H., et al. (2022). "Breathable and Skin-Conformal Electronics with Hybrid Integration of Microfabricated Multifunctional Sensors and Kirigami-Structured Nanofibrous Substrates." Advanced Functional Materials 32(32).
23. Jiang, S., et al. (2022). "A Snakeskin-Inspired, Soft-Hinge Kirigami Metamaterial for Self-Adaptive Conformal Electronic Armor." Adv Mater 34(31): e2204091.
24. Yong, K., et al. (2020). " Kirigami-inspired strain-insensitive sensors based on atomically-thin materials." Materials Today 34: 58-65.
25. Hirai, T., et al. (2022). "Elastocaloric Kirigami Temperature Modulator." Advanced Functional Materials 32(28).
26. Zhao, R., et al. (2018). "Kirigami enhances film adhesion." Soft Matter 14(13): 2515-2525.
27. He, H., et al. (2022). "Ultrastrong and multifunctional aerogels with hyperconnective network of composite polymeric nanofibers." Nat Commun 13(1): 4242.
